# Association of Primary Care Providers’ Beliefs of Statins for Primary Prevention and Statin Prescription

**DOI:** 10.1161/JAHA.118.010241

**Published:** 2019-01-25

**Authors:** Jeffrey D. Clough, Seth S. Martin, Ann Marie Navar, Li Lin, N. Chantelle Hardy, Ursula Rogers, Lesley H. Curtis

**Affiliations:** ^1^ Duke Clinical Research Institute Duke University School of Medicine Durham NC; ^2^ Department of Medicine Duke University School of Medicine Durham NC; ^3^ Department of Population Health Sciences Duke University School of Medicine Durham NC; ^4^ Department of Duke Forge Duke University School of Medicine Durham NC; ^5^ Ciccarone Center for the Prevention of Heart Disease Division of Cardiology Johns Hopkins University School of Medicine Baltimore MD

**Keywords:** guideline adherence, prevention, shared decision making, statin, Primary Prevention, Quality and Outcomes, Lipids and Cholesterol

## Abstract

**Background:**

The 2013 American College of Cardiology/American Heart Association Cholesterol Treatment Guideline increased the number of primary prevention patients eligible for statin therapy, yet uptake of these guidelines has been modest. Little is known of how primary care provider (PCP) beliefs influence statin prescription.

**Methods and Results:**

We surveyed 164 PCPs from a community‐based North Carolina network in 2017 about statin therapy. We evaluated statin initiation among the PCPs’ statin‐eligible patients between 2014 and 2015 without a previous prescription. Seventy‐two PCPs (43.9%) completed the survey. The median estimate of the relative risk reduction for high‐intensity statins was 45% (interquartile range, 25%–50%). A minority of providers (27.8%) believed statins caused diabetes mellitus, and only 16.7% reported always/very often discussing this with patients. Most PCPs (97.2%) believed that statins cause myopathy, and 72.3% reported always/very often discussing this with patients. Most (77.7%) reported always/very often using the 10‐year atherosclerotic cardiovascular disease risk calculator, although many reported that in most cases other risk factors or patient preferences influenced prescribing (59.8% and 43.1%, respectively). Of 6172 statin‐eligible patients, 22.3% received a prescription for a moderate‐ or high‐intensity statin at follow‐up. Providers reporting greater reliance on risk factors beyond atherosclerotic cardiovascular disease risk were less likely to prescribe statins.

**Conclusions:**

Although beliefs and approaches to statin discussions vary among community PCPs, new prescription rates are low and minimally associated with those beliefs. These results highlight the complexity of increasing statin prescriptions for primary prevention and suggest that strategies to facilitate standardized discussions and to address external influences on patient beliefs warrant future study.

**Clinical Perspective**

**What Is New?**

- This study demonstrated that although primary care providers hold varying beliefs about the safety and efficacy of statins for primary prevention, these beliefs are minimally associated with statin prescription.
- Rates of statin prescription were low, and primary care providers report that a high proportion of patients are unwilling to initiate statin therapy despite their recommendation.

**What Are the Clinical Implications?**

- Because guidelines for statin therapy emphasize clinician‐patient shared decision making, efforts may be needed to support clinicians in presenting consistent and accurate information.
- Given high rates of reported patient resistance to statin prescription, efforts are needed to understand external factors that influence patient decisions, particularly sources of information that are misleading or inaccurate.

## Introduction

The 2013 American College of Cardiology/American Heart Association (ACC/AHA) Cholesterol Management Guideline included major shifts in recommended approaches to primary and secondary prevention of atherosclerotic cardiovascular disease (ASCVD).^1^ The ACC/AHA guideline significantly expanded the population eligible for statin lipid‐lowering therapy for primary prevention, mainly by emphasizing recommendations based on estimated cardiovascular risk and abandoning low‐density lipoprotein cholesterol targets. One study estimated that 12.8 million additional adults would be eligible for statin therapy compared with prior guidelines.^2^ However, before initiation of statin therapy for primary prevention, the ACC/AHA guideline emphasized a clinician‐patient risk discussion. Although guidance has been published^3^ detailing elements of discussions, little is known about how such discussions are currently being implemented in clinical practice or whether specific practices are associated with statin prescription.

Although the ACC/AHA guideline expanded the population eligible for statin therapy and recommended potentially higher doses of statin therapy for primary and secondary prevention, evidence to date indicates modest adoption in clinical practice.^4^, ^5^, ^6^, ^7^ One study evaluated statin use in 161 cardiology practices before and after the ACC/AHA guideline, finding no significant difference in trends of use of moderate‐ to high‐intensity statins for eligible subgroups, with postguideline primary prevention use of 55.2% for diabetic patients and 46.9% for patients with no clinical ASCVD but elevated ASCVD risk.^8^ A study using a national database of pharmacy and medical claims found that the ACC/AHA guideline publication was associated with a decrease in statin initiation.^9^

Given that the current ACC/AHA guideline represented a substantial change in the approach to cholesterol management while also promoting individualized decision making, it is likely that clinician and patient beliefs impact decision making. The purpose of this study is to improve our understanding of the implementation of the ACC/AHA guideline from the perspective of primary care providers (PCPs), potentially helping to explain patterns of uptake as well as to identify potential barriers and solutions. We hypothesized that community PCPs hold varying beliefs of the risks and benefits of statin therapy and report varying approaches to statin risk discussions with patients. We further hypothesized that the following PCP survey responses, pertaining to recommended elements of informed discussions, would be associated with increased statin prescription for primary prevention: (1) greater reported statin efficacy; (2) fewer number of adverse effects believed to be caused by statins; (3) lower reported deviation from the guidelines because of other cardiovascular risk factors; and (4) lower reported discordance between PCP recommendations and patient preferences.

## Methods

This was a 2‐part study. The first part entailed a survey of PCPs administered between February and April 2017. The second part consisted of a retrospective analysis of patients treated during 2014 and 2015 by the surveyed PCPs. The study was approved by the Duke Institutional Review Board. The data, analytic methods, and study materials will not be made available to other researchers for purposes of reproducing the results or replicating the procedure. Survey and focus group participants gave informed consent to participate in the study. A waiver of informed consent was approved for the retrospective analysis of electronic health records.

### Study Population

The surveyed PCP population included 164 PCPs providing care in an academic, community‐based practice network in central North Carolina. We included all internal medicine physicians, family medicine physicians, nurse practitioners, and physician assistants providing primary care services to adults in all 25 continuity clinics in the network. Providers practicing in urgent care or pediatric clinics were excluded.

The analyzed patient population included patients with at least 1 outpatient visit to the surveyed PCPs between January 1, 2014, and December 31, 2015, and who met a 2013 ACC/AHA Guideline recommendation for statin therapy for primary prevention based on 10‐year ASCVD risk (≥7.5%) or diabetes mellitus diagnosis.^1^ Patients were assigned to a responsible PCP according to the hierarchical steps that were selected to identify the PCP most likely to have engaged in a statin risk discussion with patients: (1) PCP prescribing the initial statin prescription; (2) PCP ordering the first lipid panel; (3) PCP with initial annual or wellness visit during the study period; and (4) PCP with first outpatient visit. We excluded patients with any ASCVD diagnosis before or during the study period, statin prescription before January 1, 2014, low‐density lipoprotein level >189 mg/dL or <70 mg/dL, triglycerides >500 mg/dL, death during the study period, allergy to any statin, or date of birth before December 31, 1940 or after January 1, 1974.

### Survey

The survey was developed and pilot tested in 2 focus groups with a total of 13 additional PCPs who provided feedback about the survey content and understandability. We sent an introductory e‐mail to all eligible providers and mailed paper survey materials to their clinics, which participants returned via mail. We offered a $25 payment for completion of the survey. We sent 2 follow‐up e‐mails, including an electronic version of the survey to nonresponders.

Survey questions addressed several key components recommended by Martin et al for clinician‐patient risk discussions (see Data S1 for full survey).^3^ The survey included demographic questions and a question about the frequency of use of the ACC/AHA ASCVD risk calculator when discussing statin use for primary prevention. PCP respondents were asked to estimate the relative risk reduction for cardiovascular disease events from moderate‐ and high‐intensity statins. Respondents were asked questions about the overall need to discontinue statin therapy (provider or patient initiated) because of adverse effects and to indicate whether they believed statins cause key harms and how frequently they discuss individual harms with patients. Respondents were also asked to estimate how often they use additional cardiovascular risk factors when making recommendations and, finally, how often patient preferences ultimately result in a different strength or decision to initiate statin therapy than the clinician would prefer.

### Patient Data

For the patient population, we extracted their clinical data from the health system's electronic health record system spanning 2010 to 2015. This included patient demographics along with 6‐years’ worth of encounter information, visit details, diagnoses, vital signs, laboratory values, procedures, and prescriptions. These data were used to identify a patient's PCP, determine study eligibility, estimate ASCVD risk, determine statin prescription, and conduct adjusted analyses below.

### Statistical Analysis

Descriptive statistics were calculated for the PCP survey responses. Differences in each individual survey question were examined between physicians and nonphysician providers using *t* tests, Pearson χ^2^ tests, or Mantel‐Haenszel χ^2^ tests. Association between survey questions was assessed using Pearson correlations or ANOVA tests.

With the PCP survey and linked patient data together, we conducted 3‐level generalized linear mixed regression models (with logit link and binary distribution) to evaluate how the likelihood of statin prescription was affected by PCP beliefs and experiences separately for each of the 4 key survey questions. The approach accounts for the hierarchical data structure, with patients (level 1) nested in PCPs (level 2) and PCPs nested in practices (level 3). Level 1 variables include patient demographics (patient age, sex, and race/ethnicity), medical history (chronic obstructive pulmonary disease, chronic kidney disease, depression, or breast or prostate cancer), and sociodemographic variables (insurance carrier). Level 2 variables included an indicator variable as to whether the PCP was a physician or nonphysician, provider survey variables, and PCP demographic variables (years in practice and race/ethnicity). Provider's years in practice as well as patient's age were entered into models as continuous variables, whereas all others were entered as categorical variables (as shown in Tables 1 and 2). By using 2 random statements specifying practice as subject at level 3 and then provider nested within practice as subject at level 2, a random intercept was added to the model at level 2 to account for the heterogeneity among PCPs and at level 3 to account for the heterogeneity between practices. Significance of the random intercepts was evaluated by likelihood ratio statistics based on the residual pseudolikelihood. We reported odds ratio with 95% CI estimates from full model as adjusted and those from models with survey response alone as covariate as unadjusted. To examine whether a diagnosis of diabetes mellitus moderates the associations tested, a set of models were conducted with additional covariates of diabetes mellitus and its interaction with the survey variable. Odds ratios were estimated for diabetic patients. All tests are 2 sided and at 0.05 significance level without correction for multiple testing. Our data analysis was generated using SAS software, Version 9 for Linux, Copyright 2002 to 2012 by SAS Institute Inc (Cary, NC).

**Table 1:** Survey Responses by PCPs for Beliefs and Practices of Risk Discussions for Statin Therapy in Primary Prevention

Variable	All Respondents (N=72)	Providers With Eligible Patients in 2014–2015 (N=55)
Provider characteristics		
Female sex, %	66.7	60.0
Age, median (25th–75th percentile), y	44.5 (36.5–51.5)	45.0 (38.0–55.0)
Time in practice, median (25th–75th percentile), y	14.0 (5.5–20.0)	15.0 (7.0–20.0)
Race/ethnicity, %		
White	70.8	69.1
Black/African American	5.6	3.6
Asian	12.5	14.5
Other	11.1	12.8
Primary degree, %		
Doctorate of medicine or osteopathy	73.6	80.0
Nurse practitioner or physician assistant	26.4	20.0
Survey responses		
How often do you use the ASCVD risk estimator, %		
Always/very often (>75%)	77.7	76.3
Rarely/infrequently (<25%)	5.6	5.5
Estimated relative risk reduction, median (25th–75th percentile), %		
Moderate‐intensity statin	25 (15–30)	25 (15–30)
High‐intensity statin	45 (25–50)	35 (20–50)
How often do you discuss harm, %		
Incident diabetes mellitus		
Always/very often (>75%)	16.7	18.2
Rarely/infrequently (<25%)	55.6	56.3
Myopathy		
Always/very often (>75%)	72.3	67.2
Rarely/infrequently (<25%)	1.4	1.8
Rhabdomyolysis		
Always/very often (>75%)	38.9	36.4
Rarely/infrequently (<25%)	36.1	40.0
Liver injury		
Always/very often (>75%)	41.7	40.0
Rarely/infrequently (<25%)	29.2	34.5
Cognitive impairment		
Always/very often (>75%)	12.5	10.9
Rarely/infrequently (<25%)	52.7	54.5
Those indicating belief that statins cause each harm		
Incident diabetes mellitus, %	27.8	30.9
Myopathy, %	97.2	96.4
Rhabdomyolysis, %	83.3	85.5
Liver injury, %	66.7	67.3
Cognitive impairment, %	13.9	14.5
Total no. of harms	3 (2–4)	3 (2–3)
Estimated patients needing to discontinue statins, median (25th–75th percentile), %	10 (10–20)	10 (10–20)
How often do other cardiac risk factors influence statin prescribing, %		
Always/very often (>75%)	36.2	30.9
Often (50%–75%)	23.6	21.8
Sometimes (25%–50%)	25.0	27.3
Rarely/infrequently (<25%)	15.3	20.0
How often do patient preferences result in not prescribing a statin or prescribing a different dose than you would prefer, %		
Always/very often (>75%)	23.7	21.8
Often (50%–75%)	19.4	20.0
Sometimes (25%–50%)	48.6	50.9
Rarely/infrequently (<25%)	8.4	7.2

ASCVD indicates atherosclerotic cardiovascular disease; PCP, primary care provider.

**Table 2:** Patient Characteristics for Survey Respondents and Nonrespondents

Characteristics	Survey Respondent PCP	Survey Nonrespondent PCP
Patients, N	6172	10 630
Age, median (25th–75th percentile), y	61.2 (54.2–66.6)	62.2 (55.6–67.2)
Female sex, %	43.7	41.5
Hispanic, %	1.4	1.5
Non‐Hispanic black, %	35.9	29.2
Diabetes mellitus, %	37.0	36.0
Chronic obstructive pulmonary disease, %	2.8	2.8
Chronic kidney disease, %	5.0	5.6
Depression, %	9.6	8.6
Prostate/breast cancer, %	1.8	1.8
Total cholesterol, median (25th–75th percentile), mg/dL	196 (173–220)	196 (174–220)
HDL, median (25th–75th percentile), mg/dL	46 (38–56)	46 (39–56)
LDL, median (25th–75th percentile), mg/dL	120 (100–141)	119 (100–141)
Systolic blood pressure, median (25th–75th percentile), mm Hg	132 (122–144)	132 (122–142)
Treated for hypertension, %	53.7	48.7
Current smoker, %	8.7	5.4
ASCVD 10‐y risk score, median (25th–75th percentile), %	11.4 (8.7–16.5)	11.5 (8.8–16.5)
Framingham 10‐y risk score, median (25th–75th percentile), %	7.5 (3.4–12.2)	8.3 (3.7–12.4)
Eligible for statin based on ATP‐III criteria, %	40.7	40.1
Prescription of moderate‐ or high‐intensity statin during 2014–2015, %	22.3	19.0

ASCVD indicates atherosclerotic cardiovascular disease; ATP‐III, Adult Treatment Panel III; HDL, high‐density lipoprotein; LDL, low‐density lipoprotein; PCP, primary care provider.

### Results

Table 1 summarizes survey responses. Seventy‐two PCPs (43.9%) completed the survey. Most were women (66.7%), white (70.8%), and physicians (73.6%), with median time in practice of 14 years (interquartile range, 5.5–20 years). Of the 72 who completed the survey, 55 had assigned patients during 2014 to 2015 and were included in the electronic health record data analysis.

Providers most often estimated that moderate‐ and high‐intensity statins lowered ASCVD risk by 30% and 50%, respectively (Figure). However, small numbers of providers quoted extremely high and low estimates of effectiveness, with 8 providers estimating that statins had no effect on the risk of cardiovascular events. Provider median estimates of the ASCVD relative risk reduction for moderate‐ and high‐intensity statins were 25% (interquartile range, 15%–30%) and 45% (interquartile range, 25%–50%), respectively (Table 1).

**Figure 1:**
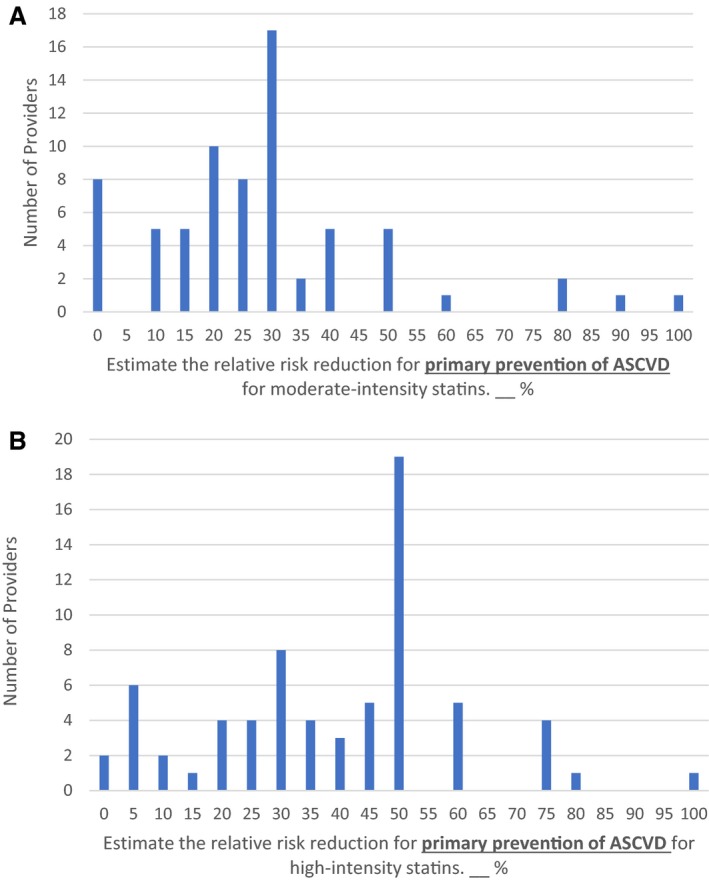
Estimated relative risk reduction for primary prevention of atherosclerotic cardiovascular disease (ASCVD) for moderate‐ and high‐intensity statins. **A**, Moderate‐intensity statin. **B**, High‐intensity statin.

Provider‐reported beliefs in harms and reported frequency of discussing harms were highly variable. Although most PCPs (97.2%) reported believing that statins cause myopathy, only 72.3% reported always or very often discussing it with patients (Table 1). Fewer providers believed statins caused diabetes mellitus (27.8%), and only 16.7% reported always (>90% of the time) or very often (75%–90% of the time) discussing this risk with their patients. Providers who believed that statins caused liver injury or cognitive impairment were more likely to report discussing these harms (*P*=0.001 and *P*=0.002, respectively). The median estimate of the percentage of patients needing to discontinue a statin because of real or perceived adverse effects was 10% (interquartile range, 10%–20%).

Although 77.7% of PCPs reported always or very often calculating 10‐year ASCVD risk, 59.8% and 43.1% reported that, in most cases, prescriptions were often, very often, or always influenced by other clinical risk factors or patient preferences, respectively (Table 1).

Results were similar comparing physicians with nonphysician providers (data not shown) for all questions, except that physicians were more likely to indicate that they believe statins cause incident diabetes mellitus (physicians: 37.7% yes, 5.7% no, and 56.6% unsure or evidence not definitive; nonphysicians: 0% yes, 31.6% no, and 78.4% unsure or evidence not definitive; *P*=0.002). Provider beliefs in the risks and benefits of statin therapy were not statistically significantly correlated with each other or the use of additional clinical factors in making recommendations. There was a modest positive correlation (*r*=0.28, *P*=0.016) between the likelihood of using other clinical risk factors and the influence of patient preferences.

### Association Between Survey Responses and Statin Prescription

Table 2 presents data for patients assigned to survey respondent PCPs and PCPs who did not complete the survey. The proportion of patients assigned to PCPs through each step in the alignment algorithm was: 20.2% based on statin prescription, 60.0% based on ordering of a lipid panel, 11.7% for the first annual or wellness visit, and 8.2% for the first outpatient visit. Generally, patient characteristics were similar between the 2 groups. Among patients included in the analysis, the mean age was 59.9 years, 43.7% were women, 35.9% were non‐Hispanic black, 37.0% had diabetes mellitus, the mean 10‐year ASCVD risk score was 13.5% (SD, 7.8%), and 40.7% were eligible for statins based on Adult Treatment Panel III criteria. A total of 22% of these patients received a prescription for a statin during the study period compared with 19% of patients assigned to nonrespondent PCPs.

Table 3 includes the unadjusted and adjusted odds ratios for receiving a moderate‐ or high‐intensity statin during the study period based on incremental response. There was no association between statin prescription and PCP beliefs in the benefits or harms of statin therapy for primary prevention (as measured by estimated relative risk reduction of ASCVD events for high‐intensity statins and cumulative harms believed to be caused by statins). Reported influence of other cardiac risk factors and patient preferences had a modest association with statin prescription in unadjusted analyses, but after modeling to account for patient and provider characteristics, only a reported decreased use of risk factors beyond estimated ASCVD risk was associated with a modest increased likelihood of statin prescription (odds ratio, 1.12; 95% CI, 1.03–1.23). Among diabetic patients, both a decreased use of other risk factors and decreasing proportion of patient preferences deviating from provider recommendations were modestly associated with statin prescription (odds ratios, 1.19 [95% CI, 1.07–1.32] and 1.25 [95% CI, 1.11–1.42], respectively). The variance component of the intercept in every model was statically significant, indicating that significant heterogeneity among providers or patients remained after adjusting for the covariates included in the models.

**Table 3:** Association Between Survey Responses and Statin Prescription

Variable	Odds Ratio (95% CI) of Statin Prescription Based on Incremental Response		
	Overall	Patients With Diabetes Mellitus	Patients Without Diabetes Mellitus
Statin efficacy^a^			
Unadjusted	1.01 (0.98–1.03)	1.01 (0.98–1.03)	1.01 (0.98–1.04)
Adjusted	1.02 (0.99–1.04)	1.02 (0.99–1.05)	1.01 (0.98–1.04)
Cumulative statin harms^b^			
Unadjusted	1.00 (0.94–1.06)	1.03 (0.98–1.11)	0.98 (0.91–1.05)
Adjusted	1.03 (0.96–1.10)	1.07 (0.99–1.15)	0.99 (0.92–1.07)
Other risk factors^c^			
Unadjusted	1.13 (1.05–1.23)^d^	1.19 (1.08–1.30)^d^	1.08 (0.98–1.18)
Adjusted	1.12 (1.03–1.23)^d^	1.19 (1.07–1.32)^d^	1.05 (0.95–1.17)
Patient preferences^e^			
Unadjusted	1.12 (1.03–1.23)^d^	1.26 (1.13–1.39)^d^	0.98 (0.89–1.09)
Adjusted	1.09 (0.98–1.21)	1.25 (1.11–1.42)^d^	0.97 (0.86, 1.10]

a Statin efficacy was measured by estimated relative risk reduction for high‐intensity statins.

b Cumulative statin harms was the number of harms each provider believed to be caused by statins.

c Other risk factors was the reported percentage of cases (ordered by reduced frequency) that other risk factors influenced statin recommendations.

d *P*<0.05.

e Patient preferences is the reported percentage of cases (ordered by reduced frequency) that patient preferences alter prescription from primary care providers’ preference.

## Discussion

We found that PCPs in our study sample held varying beliefs about the risks and benefits of statin therapy for primary prevention and, therefore, varied in their approach and experience discussing this treatment with patients. Variation was most pronounced for belief and discussion of potential harms, consideration of risk factors other than ASCVD risk, and the degree to which patient preferences affected decisions to prescribe. However, consistent with other studies, rates of statin initiation for eligible patients were low and minimally associated with PCP beliefs or experience.

PCPs in our study sample reported commonly using the ASCVD risk calculator, indicating familiarity and at least partial adoption of the ACC/AHA guideline. Estimates of the relative risk reduction of statins were close to those supported by the literature and much more accurate than other physician estimates of common primary care interventions.^10^ Although estimates derived from patient‐level meta‐analyses note that the degree of relative risk reduction is proportional to the amount of low‐density lipoprotein lowering on therapy, 30% to 50% are commonly cited figures for an average patient response.^3^, ^11^, ^12^ Some respondents indicated extreme effects of up to 100% relative risk reduction, which may have reflected a misunderstanding of the question despite provision of an example.

PCPs varied in their reported frequency of discussing potential adverse effects as well as their reported beliefs about the strength of evidence that statins cause adverse effects. These conversations may be complex and time‐consuming because a PCP may need to discuss the nature of the adverse effect, the likelihood of its occurrence, and the strength of evidence. Myopathy was almost universally believed to be an adverse effect caused by statins and typically discussed. However, there may be significant heterogeneity in how myopathy and all muscle‐related symptoms are discussed, including the likelihood that statins cause these symptoms. Evidence from placebo‐controlled randomized trials has demonstrated that most symptomatic adverse events attributed to statin therapy in routine practice are not caused by it and severe cases of myositis are rare.^13^ Conversely, although risk of incident diabetes mellitus was emphasized in the ACC/AHA guideline,^1^ this risk was infrequently discussed and most providers did not believe statins cause diabetes mellitus. This may be partially explained by the fact that although statins may accelerate a diagnosis of diabetes mellitus, they have not been associated with an increased risk of microvascular or macrovascular complications and so the clinical significance is unclear.^3^ Because adverse effects are typically cited as a reason by patients for not taking or discontinuing statins,^14^ this is likely to be a critical component of informed discussions and warrants further study. The median estimate of 10% of patients needing to discontinue statins because of adverse effects is much lower than reported rates of discontinuation, which have been as high as half of all patients at 1 year.^15^, ^16^ It is possible that this reflects a perception that a minority of patients actually experience adverse events. Given high reported rates of patient preferences influencing decisions, it is also possible that statins are being selectively targeted to patients who are inclined to continue therapy.

The overall rate of statin initiation for eligible patients who were not receiving therapy already was low, at 22.3%, for surveyed PCPs. Studies that have demonstrated changes in behavior after release of the guidelines have most commonly demonstrated a shift to higher‐intensity statins among patients with clinical ASCVD, particularly among patients undergoing percutaneous coronary intervention.^17^ It is likely that a different set of factors influence prescription for primary prevention, particularly the decision to initiate treatment among patients who were not previously eligible or receiving statin therapy. PCPs in our survey reported high rates of at least partial guideline adoptions (ie, calculating ASCVD risk) as well as beliefs in statin efficacy and harms consistent with and in many cases favorable in comparison to those reported in the literature. Combined with high rates of reported patient discordance with provider recommendations, these data suggest that efforts to increase statin prescription may benefit from understanding factors influencing patients’ beliefs both in and out of the clinic, rather than targeting provider motivation or knowledge.

Efforts to increase statin prescription must balance the need to respect patient autonomy. As an example, the Centers for Medicare and Medicaid Services’ Million Hearts Cardiovascular Disease Risk Reduction Model rewards practices for risk assessment, engaging in shared decision making, and ultimately reducing cardiovascular risk.^18^ Incentives toward shared decision making and risk reduction may conflict when a patient prefers to forego treatment. Although this model targets a high‐risk population (ASCVD at least 30%), extension of similar policies to lower‐risk populations may heighten the conflict, as evidenced by the lower prescription rates in the lower‐risk subpopulations in this study.

On November 10, 2018, the ACC and AHA along with multiple additional societies released an updated guideline for the management of blood cholesterol.^19^ This guideline continues to recommend a clinician‐patient risk discussion when considering statins for primary prevention and further emphasized the importance of personalized risk assessment and shared decision making to initiate therapy. The heterogeneity in clinician beliefs and approaches identified in our study suggests that patients are at risk for receiving different information and recommendations from different clinicians. Approaches to help standardize these discussions to ensure that personalized decisions comport with patient preferences warrant further study. In addition, the overall low rates of prescription, limited influence of clinician responses on prescribing, and high reported rates of discordance between clinician recommendations and patients’ acceptance of prescription indicate a need for further understanding of external factors and information sources that influence patient beliefs. Approaches to address inaccurate information or undesirable influences may be needed.

Our study is not without limitations. We surveyed providers about their practices yet did not directly observe the content of provider discussions with patients or frequency of shared decision making. It is also possible that views may have changed between the retrospective study period and the survey. The period of analysis was limited to 2014 to 2015 to avoid the potential impact of the US Preventive Services Task Force draft recommendation for the use of statins for primary prevention in adults.^20^ If providers’ views converged over time, as might be expected with national attention and educational activities catalyzed by the guidelines, our study may underrepresent variability in views immediately after release and could have overestimated the effect of providers’ views on statin prescription. If there was a net divergence or other significant change, we may have failed to detect a meaningful association between views and statin prescription. However, this would not affect the overall findings that views and reported approaches were variable in 2017 and that there was an overall low rate of statin prescription after release of the guidelines.

Next, this study was limited to a single health system, which may underestimate variability in provider beliefs, although the providers in this study did practice at 25 different clinics, which serve a diverse population. Finally, our response rate was 43.9%, which may limit generalizability of the responses; however, patient characteristics and statin prescription rates were similar among responders and nonresponders.

## Conclusion

Although community‐based PCPs often accurately estimate the benefit of statin therapy, beliefs in potential adverse effects and reported content of clinician‐patient discussions vary. In addition, statin prescription rates for primary prevention are low and minimally influenced by these beliefs, suggesting a need to address patient‐level factors that may influence decisions to initiate statin therapy.

## Sources of Funding

This work was supported by American Heart Association Grant 16MCPRP30420003. Dr Navar is supported by a K‐award from the NIH. Her effort for this study was not directly supported by the AHA award.

## Disclosures

Martin has received consulting fees from Sanofi/Regeneron, Amgen, and Quest Diagnostics (all modest) and is the coinventor of a method of assessing low‐density lipoprotein cholesterol levels filed by his institution. Navar has received research funding from Amgen, Sanofi, and Regeneron (all significant) and consulting fees from Amgen and Sanofi (all modest). The remaining authors have no disclosures to report.

## Supporting information


**Data S1.** Survey of Primary Care Clinician Beliefs and Approaches to Statin Therapy.
