# Supplementary material for: Association of Primary Care Providers’ Beliefs of Statins for Primary Prevention and Statin Prescription
Source: J Am Heart Assoc. 2019 Jan 25;8(3):e010241. doi: 10.1161/JAHA.118.010241 (PMC6405576; doi:10.1161/JAHA.118.010241)
Supplement: Supplementary file 1 — Data S1. Survey of Primary Care Clinician Beliefs and Approaches to Statin Therapy. [file JAH3-8-e010241-s001.pdf]

Supplemental Material

Data S1.

Survey of Primary Care Clinician Beliefs and Approaches to Statin Therapy

Statement on Consent: By completing and returning this study I acknowledge that I have reviewed the informed consent form and agree to participate in this study.

Section 1: Provider Characteristics. Please select the BEST response for each question.

1. Please state your full name: ____
2. Please list any previous names since 2010: ____
3. Please indicate your sex:
    a. Male
    b. Female
4. Please indicate your current age in years ____
5. How many years have you been in clinical practice? ____
6. Please indicate your race:
    a. American Indian/Alaska Native
    b. Asian
    c. Black/African-American
    d. Native Hawaiian/Pacific Islander
    e. White
    f. Other (specify) ____________________________
    g. Prefer not to respond
7. Please indicate your ethnicity:
    a. Hispanic or Latino
    b. Not Hispanic or Latino
    c. Prefer not to respond
8. Please indicate your primary degree
    a. Doctor of Medicine (MD)
    b. Doctor of Osteopathic Medicine (DO)
    c. Nurse Practitioner (NP)
    d. Physician Assistant (PA)
    e. Other

Section 2: Statin Therapy for Primary Prevention. Please select the BEST response for each question.

1. How often do you use the ACC/AHA atherosclerotic cardiovascular disease (ASCVD) risk estimator when discussing statin therapy **for primary prevention?**

    a. Always (>90%)
    b. Very Often (75-90%)
    c. Often (50-75%

d. Sometimes (25-50%)
e. Rarely (<25%)

2. Indicate how often you discuss EACH of the following harms when considering statin therapy for primary prevention.

	Rarely	Infrequently	Sometimes	Often	Very Often	Always
a. Incident diabetes	__	__	__	__	__	__
b. Myopathy	__	__	__	__	__	__
c. Rhabdomyolysis	__	__	__	__	__	__
d. Liver injury	__	__	__	__	__	__
e. Cognitive impairment	__	__	__	__	__	__

3. Indicate whether you think that statins cause EACH of the following harms based on your experience and the scientific literature.

	No	Yes	Evidence is not definitive	Not sure
a. Incident diabetes	__	__	__	__
b. Myopathy	__	__	__	__
c. Rhabdomyolysis	__	__	__	__
d. Liver injury	__	__	__	__
e. Cognitive impairment	__	__	__	__

4. How often would you estimate a statin needs to be discontinued (including drug holidays), given patient complaints or adverse medical events?

_______%

5. Certain clinical cardiac risk factors are not included in the ACC calculator (examples: family history, LDL levels, C-reactive protein, coronary calcium score, ankle-brachial index and other comorbidities).

   How often do these traditional risk factors influence your statin prescribing?

   a. Always (>90%)
   b. Very Often (75-90%)
   c. Often (50-75%)
   d. Sometimes (25-50%)
   e. Infrequently (10-25%)
   f. Rarely (<10%)

6. How often do patient preferences ultimately result in you either not prescribing a statin or prescribing a different dose than you would prefer? Circle the best answer.

   a. Always (>90%)
   b. Very Often (75-90%)
   c. Often (50-75%)
   d. Sometimes (25-50%)
   e. Infrequently (10-25%)
   f. Rarely (<10%)

7. Estimate the relative risk reduction for **<u>primary prevention of ASCVD</u>** for each class of statins.

Example: For a patient with a baseline 10-year risk for ASCVD of 10%, a 50% relative risk reduction would result in a 5% 10-year risk for ASCVD

a. Moderate intensity statin ______%

b. High intensity statin ______%

Moderate Intensity Statins	High Intensity Statins
Rosuvastatin 5-10 mg<br>Atorvastatin 10-20 mg<br>Simvastatin 20-40 mg<br>Pravastatin 40-80 mg<br>Lovastatin 40 mg<br>Fluvastatin 80 mg<br>Pitavastatin 2-4 mg	Rosuvastatin 20-40 mg<br>Atorvastatin 40-80 mg

8. Please provide any additional comments below:
